# Interactions between Reduced Graphene Oxide with Monomers of (Calcium) Silicate Hydrates: A First-Principles Study

**DOI:** 10.3390/nano11092248

**Published:** 2021-08-31

**Authors:** Mohammadreza Izadifar, Jorge S. Dolado, Peter Thissen, Andres Ayuela

**Affiliations:** 1 Karlsruhe Institute of Technology, Institute of Functional Interfaces, 76344 Eggenstein-Leopoldshafen, Germany; mr.izadifar@icloud.com (M.I.); peter.thissen@kit.edu (P.T.); 2 Institute of Construction and Building Materials, Technical University of Darmstadt, Franzizka-Braun-Str. 3, 64287 Dramstadt, Germany; 3 Centro de Física de Materiales-Materials Physics Center, Centro Mixto CSIC-UPV/EHU, 20018 San Sebastián, Spain; Jorge_dolado002@ehu.eus; 4 Donostia International Physics Center, 20018 San Sebastián, Spain

**Keywords:** composite material, calcium silicate hydrate, interlayer microstructure, nanomaterials, DFT calculations, reduced graphene oxide

## Abstract

Graphene is a two-dimensional material, with exceptional mechanical, electrical, and thermal properties. Graphene-based materials are, therefore, excellent candidates for use in nanocomposites. We investigated reduced graphene oxide (rGO), which is produced easily by oxidizing and exfoliating graphite in calcium silicate hydrate (CSHs) composites, for use in cementitious materials. The density functional theory was used to study the binding of moieties, on the rGO surface (e.g., hydroxyl-OH/rGO and epoxide/rGO groups), to CSH units, such as silicate tetrahedra, calcium ions, and OH groups. The simulations indicate complex interactions between OH/rGO and silicate tetrahedra, involving condensation reactions and selective repairing of the rGO lattice to reform pristine graphene. The condensation reactions even occurred in the presence of calcium ions and hydroxyl groups. In contrast, rGO/CSH interactions remained close to the initial structural models of the epoxy rGO surface. The simulations indicate that specific CSHs, containing rGO with different interfacial topologies, can be manufactured using coatings of either epoxide or hydroxyl groups. The results fill a knowledge gap, by establishing a connection between the chemical compositions of CSH units and rGO, and confirm that a wet chemical method can be used to produce pristine graphene by removing hydroxyl defects from rGO.

## 1. Introduction

Graphene [1] is a two-dimensional honeycomb plane of sp^2^ carbon atoms, and has received considerable attention for its use in applications such as electronic devices [2], energy storage devices [3], and composite materials [4,5], because of its unique mechanical [6,7], electronic [8], thermal [9,10,11,12], and chemical properties [13,14,15,16,17]. The development of a cheap method of fabrication of high-quality graphene remains a considerable challenge [15]. Graphene can be produced by chemical vapor deposition (CVD), using a catalytic metal substrate made of materials such as Cu or Ni [18,19], or by mechanical and chemical exfoliation of graphite, with deposition of the exfoliated pieces on various substrates [1,15,20]. The chemical vapor deposition method involves the use of high-temperature procedures and specialist equipment, and is a relatively expensive method of producing graphene with very few defects. A great deal of attention has been paid to the development of methods involving the chemical oxidation and exfoliation of graphite, to yield graphene oxide (GO), with the subsequent reduction in the oxygen-containing functional groups, using thermal, chemical, or electrochemical reduction methods, to yield reduced graphene oxide (rGO), because such methods are solution-based [21,22]. Therefore the procedure for fabricating rGO is briefly described.

**Figure 1:**
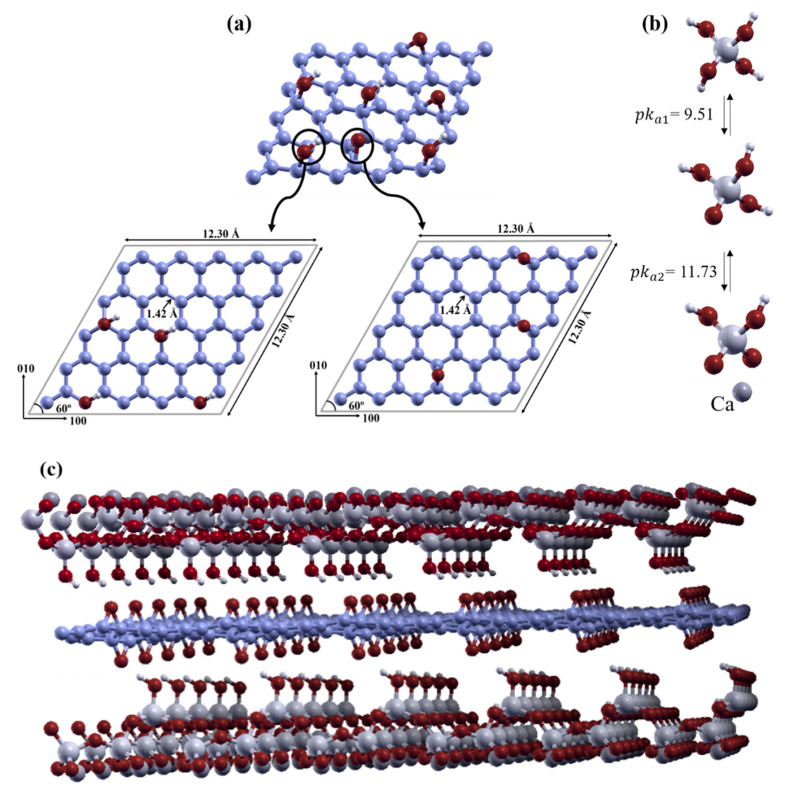
(**a**) Reduced graphene oxide with hydroxyl and epoxide groups. The lower, left- and right-hand panels show hydroxyl and epoxide surface models, respectively. (**b**) The *pk* ratios for calcium silicate hydrate gels. (**c**) Calcium silicate hydrate composites consisting of calcium silicate hydrate gel and epoxide/reduced graphene oxide. Carbon atoms are indicated in blue; oxygen, in red; hydrogen, in white; silicon, in light grey; and calcium, in dark grey.

The most widely used technique for preparing graphite oxide by oxidizing graphite is known as Hummer’s method [20,23,24,25]. The oxidization of graphite is an effective way of increasing the interlayer distance between the graphene sheets by adding oxygen-containing functional groups, such as hydroxyl, epoxide, carboxyl, and carbonyl groups, to facilitate the exfoliation [22]. The exfoliation of graphite oxide is an important process for producing graphene oxide (GO), as an intermediate between graphite and graphene. Graphite oxide can be completely exfoliated into GO by mechanically stirring a water and graphite oxide mixture for a long time [26], or by ultrasonicating graphite oxide in a polar organic solvent or aqueous solution [14,27]. The basal plane of GO is mostly occupied by epoxide and hydroxyl groups, but the edge plane is mostly occupied by carbonyl and carboxyl groups [28]. Hydroxyl and epoxide functional groups can be considered to be surface structural defects in the graphene layer, as shown in Figure 1a. There are a few functional groups at the edge of defective graphene, so our work will concentrate on the surfaces, which have many epoxide and hydroxyl groups [29].

Many recent studies have focused on manufacturing very pure rGO sheets (i.e., graphene with low coverage densities of oxygen-containing functional groups) from GO [22]. Feng et al. produced rGO structures with low oxygen contents (down to 5.6% by weight), using Na and NH_3_ treatments with active solvated electrons as a strong reducing agent [30]. Liao et al. [31] produced graphene from exfoliated graphite oxide in deionized water at ~pH 3, between 12 and 48 h at 120 or 95 °C, at an oxygen-to-carbon reduction ratio (O:C) of about 1:6, and analyzed the product by means of C^1s^ X-ray photoelectron spectroscopy spectra. By considering Fourier transform infrared spectra, they found a marked decrease in the number of hydroxyl and epoxide groups. Pei et al. [32] fabricated very conductive and flexible graphene films by reducing GO films, by immersion in a solution of HI, an 85% N_2_H_4_·H_2_O solution, and a 50 mM NaBH_4_ aqueous solution at room temperature. They found that after reducing the GO films with HI, most of the oxygen-containing groups from the GO film had been removed, causing the C–C bonds to become dominant, giving a C/O atom ratio ≥ 12 and an electrical conductivity of up to 298 S/cm. Much lower C/O atom ratios and electrical conductivities were found for higher GO films that were reduced using N_2_H_4_·H_2_O and NaBH_4_.

Concrete- and cement-based materials are only second to water, in terms of their use around the world [33,34,35,36]. The total annual concrete production around the world is ≥20 × 10^9^ t and it is currently increasing by 5% per year, and contributes 5–10% to global anthropogenic carbon dioxide emissions [37,38]. The chemically active ingredients of cements in clinker particles are hydrated to produce cement paste. Unhydrated clinker and calcium silicate hydrates form a multi-scale porous composite, the primary binding phase of which is known as CSH gel [39,40]. This acts as a glue that adheres to fine and coarse aggregates to create concrete [37,41]. CSH gel has a complex structure [33,42,43], including water in the interlayers, layered material structures at the nanoscale [44,45], a globular texture at the mesoscale [46,47,48], and a multi-scale porous structure [49,50,51]. The engineering properties of cement-based materials are largely controlled by the properties of the CSH gel [33,39,52], which has an intrinsically brittle nature and is weak in tension. This weakness is usually overcome by reinforcing cement-based materials with metal fibers and, more recently, the possibilities offered by inorganic or carbon nanotubes are being explored [53,54,55,56]. In the latter case, it is relatively difficult to combine the aqueous solution involved in the cementitious matrix with the hydrophobic carbon nanostructures, such as pristine carbon nanotubes. An understanding of the defects seems to be key in controlling the final properties of the cementitious nanocomposite [56].

The current main challenge in the field area lies in improving our understanding of the mechanisms involved in the interactions between the chemical components of cementitious CSH gel moieties [57] and pristine or defect-containing graphene sheets in water, at the nanoscale. This is particularly relevant in the context of Dimov et al. [58], who demonstrated, experimentally, that graphene-enabled nanoengineered concrete composites can have ultra-high strengths and interesting additional functionalities. Yao et al. recently synthesized very tough highly ordered CSH–GO composites, assuming that they contain COOH groups [59]. Hou et al. recently used reactive force field molecular dynamics to investigate the mechanical properties and reactivities of GO sheets that are functionalized with hydroxyl (C–OH), epoxy (C–O–C), carboxyl (COOH), and sulphonic (SO_3_H) groups, with a 10% ultra-confined coverage with the calcium silicate hydrate gel (CSH), as shown in Figure 1b [60]. Calculations using potentials have mainly been performed to study the GO–COOH groups on graphene, but such groups are attached to the GO edges, so constitute a minority of the groups attached to GO. We therefore focus on hydroxyl and epoxy groups.

To our best knowledge, the mechanism involved in the interactions between rGO and monomers in CSH gels during the fabrication of cementitious composite materials (Figure 1c) have not been studied previously. The aim of this study was to improve our fundamental understanding of the interactions between CSHs (e.g., CSH gel) with rGO, using density functional theory (DFT) calculations. Interactions at the interface were taken into account by calculating the adsorption energies of optimized CSH gel units with OH/rGO and epoxide/rGO sheets in various initial configurations. The results fill a key knowledge gap, by establishing connections between the chemical components of CSH gel in cementitious materials and rGO.

## 2. Methods and Computational Models

### 2.1. Simulation Parameters

The interactions between rGO and CSHs are studied by performing DFT electronic structure calculations [61]. The Vienna ab initio simulation package [62,63,64] and the projected augmented-wave method were used to define electron–ion interactions. A well-converged plane-wave cutoff energy of 400 eV was employed. The electron exchange and correlation functional was used in the generalized gradient approximation with Perdew−Burke−Ernzerhof parametrization [65]. A force tolerance of 0.01 eV/Å was used for the structural optimizations. The Brillouin zone was sampled using a well-converged k-sampling, given by 2 × 2 × 1 Monkhorst-Pack k-points for the whole system [66]. The density of state (DOS) was calculated using a refined mesh of 34 × 34 × 1 Monkhorst-Pack k-points. The charge transfers were calculated using Bader analysis, with the code developed by Henkelman et al. [67].

### 2.2. Model Building

Models of rGO, focusing on hydroxyl or epoxide groups, are developed with periodic boundary conditions in the x- and y-directions, to remove finite length effects, and a well-converged vacuum slab 10 Å thick to avoid interactions with adjacent cells in the z-direction. Larger boxes of 15 Å were also tested for some cases involving rGO, Ca, and silicate units, and the calculated energy differences were found to converge at the sub meV level. The optimized primitive rGO unit cell therefore has the parameters a = 12.30 Å, b = 12.30 Å, c = 10 Å, α = 90°, β = 90°, and γ = 60°. We treated silicate hydrate moieties as Si(OH)_4_ monomers, because these moieties occur in solution when the calcium silicates in cementitious clinkers are hydrated. SiO(OH)_3_^−^ units that were produced in small amounts during the hydration process, causing the pH of cement, were also studied. The contributions of van der Waals interactions were not considered in all the configurations, because the differences in van der Waals potential energies were found to be small when calculated in some tests. The ΔE was calculated from the difference between the energies of the relaxed configuration and the ground-state structure. Further, rGO structures with oxygen-to-carbon reduction ratios (O:C) of 1:50 were used in the simulation models. (The vacancy–adatom pair is one of the most common defects in graphene, but the defect mobility is high [68]. Thus, single vacancies would be saturated in solutions before entering the composites).

### 2.3. Adsorption Energy

The adsorption energy (E_ads_) relates to interactions between the sorbent and substrate, and was calculated using Equation (1), as follows:

E_ads_ = E_Total_ − (E_Sub_ + E_Ab_) (1)

where E_Total_ is the total energy of the composite systems, E_Sub_ is the energy of the graphene or rGO plane, and E_Ab_ is the energy of the absorbed moiety [69].

## 3. Results

### 3.1. Hydroxyl/rGO with Silicate Hydrate Moieties

#### 3.1.1. Hydroxyl/rGO with Si(OH)_4_ Silicate Hydrate Units

**Figure 2:**
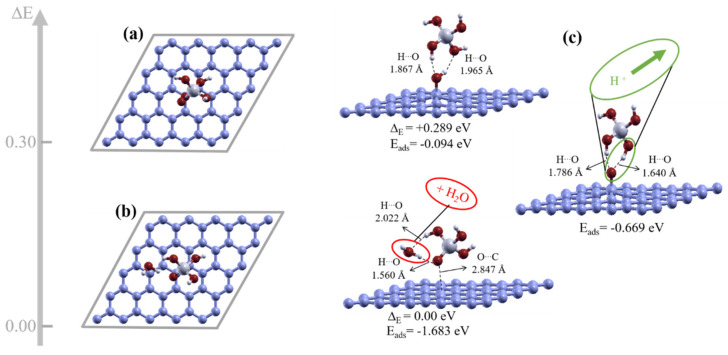
Top and side views of the geometric configurations obtained for the initial geometric structure of Si(OH)_4_ on a hydroxyl/reduced graphene oxide (rGO) sheet. (**a**) Expected optimized physisorbed configuration. (**b**) Ground state obtained as an optimized geometric structure including a water molecule and a SiO(OH)_3_ unit on the graphene plane. (**c**) Optimized geometric structure obtained for SiO(OH)_3_ on a hydroxyl/rGO sheet for a singly negatively charged system. The energy difference ΔE and adsorption energies for Si(OH)_4_ and SiO(OH)_3_ are indicated below the respective configurations.

Feasible configurations of hydroxyl- and epoxide-reduced graphene with CSH gel moieties were calculated to allow the investigation of the mechanisms involved in the interactions between the different moieties. We were interested in investigating the mechanism involved in the interaction between Si(OH)_4_ hydrated silicate monomers and hydroxyl/rGO. Two separate configurations for our simulation models, with Si(OH)_4_ in two different positions relative to the hydroxyl/rGO sheets, as input geometric structures, were therefore first prepared. Two dissimilar Si(OH)_4_ unit configurations, with respect to the distance to the hydroxyl/rGO sheet, were found. There was a stable configuration in which the starting geometry did not change much after optimization, as shown in Figure 2a. However, optimization indicates that the ground-state structure could become chemically reconstructed, and produce a water molecule between the graphene layer and the SiO(OH)_3_ unit, as shown in Figure 2b. The energy of the reconstructed structure (Figure 2b) was ≅0.3 eV lower than the expected optimized configuration (Figure 2a). This structure corresponds to the ground-state energy structure for Si(OH)_4_ deposited slightly further from the hydroxyl/rGO sheet than in the expected configuration.

The calculated adsorption energies for the SiO(OH)_3_ and Si(OH)_4_ configurations were −1.683 eV and −0.094 eV (2.17 kcal/mol), respectively. Therefore, the SiO(OH)_3_ unit in the ground-state structure had a lower adsorption energy than the other configurations, indicating that the system containing SiO(OH)_3_ with a water molecule next to a graphene plane was the most favorable configuration. Importantly, a condensation reaction occurs when a hydroxyl group at the GO interface becomes dissociated and combines with a hydrogen atom released by Si(OH)_4_.

Then, several configurations of water molecules in a system with a SiO(OH)_3_ unit on graphene were considered, to determine the optimum location of the water molecule. The geometric structure consisting of a system containing SiO(OH)_3_, a water molecule, and graphene, shown in Figure 2b, was the ground-state structure and the most stable configuration. The ground-state structure forms for two reasons. The water molecule establishes two strong O–H hydrogen bonds (short) with SiO(OH)_3_, and, most importantly, it is near the graphene surface, implying an interaction with the graphene surface and an increase in the bonding energy.

A SiO(OH)_3_ unit on hydroxyl/rGO, in a singly negatively charged system, was also considered. Perhaps surprisingly, we found that in the ground-state structure, hydrogen is dissociated from the hydroxyl/rGO surface and is being transferred to the SiO(OH)_3_ unit to saturate the dangling oxygen atom, as shown in Figure 2c. SiO(OH)_3_ on hydroxyl/rGO in a neutral system was placed as another initial simulation model, and we also found that the hydrogen becomes dissociated from the hydroxyl/rGO surface and is transferred to the SiO(OH)_3_ unit. This indicates that a hydrogen atom becomes dissociated from the hydroxyl/rGO surface regardless of whether the system is charged or neutral. A dangling oxygen bond therefore points towards the graphene plane and is almost fully occupied through charge transfer from the graphene sheet.

#### 3.1.2. Model Calculations for an SiO(OH)_3_ Unit on Graphene: Chemisorbed and Physisorbed Configurations

**Figure 3:**
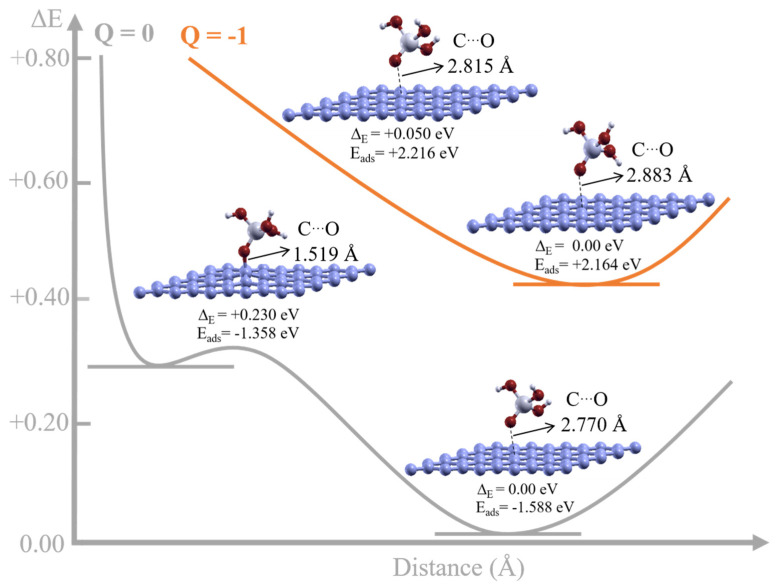
Energy scheme for the relaxed geometric structures of SiO(OH)_3_ units on graphene. Two different initial chemisorbed and physisorbed configurations with different distances between the graphene plane and SiO(OH)_3_ were found for the neutral systems, and physisorbed configurations were found for the singly negatively charged system. The energy difference ΔE (in eV) for each configuration for each charge state is given below the respective structure. The adsorption energy with respect to the SiO(OH)_3_ units is also shown in each case.

Next, we considered the adsorption properties of a SiO(OH)_3_ unit on the graphene surface. The unit on the graphene plane was placed at several distances in four different configurations, with the system being either neutral or singly negatively charged. All four configurations were optimized, and the ground-state structure was found for the configuration associated with the unit that was physisorbed to the graphene sheet. The configurations for the neutral cases are shown in Figure 3. The ground-state structure was found to be SiO(OH)_3_ physisorbed to the graphene sheet, which has a lower energy state (−0.23 eV) than the chemisorbed configuration. When the system is singly negatively charged, both relaxed structures have the SiO(OH)_3_ unit physisorbed to the graphene sheet.

The calculated adsorption energies for SiO(OH)_3_, for the physisorbed ground-state structures and the next chemisorbed configuration for the neutral system, are −1.59 and −1.36 eV, respectively, as shown in Figure 3, for the two clearly different distances. In contrast, the adsorption energies for the two similar physisorbed configurations on the negatively doped graphene plane are 2.16 and 2.22 eV, as shown in Figure 3. An electron is transferred to SiO(OH)_3_ to form SiO (OH)_3_^−^ for the total charged system, so the initial energy state must change by the difference between the neutral and charged silicate units. We expect that the adsorption of SiO(OH)_3_ to the doped graphene sheet in the neutral and singly negatively charged state is almost the same, because the doping electron must be shared between a large number of carbon atoms. The corrected adsorption energy for the SiO (OH)_3_^−^ unit, typical in a basic solution, was found to be exothermic by −1.4 eV, because charge fills the oxygen levels. It seems that the adsorption of silicate hydrate units onto rGO would remain favorable at basic pH values and with negatively charged units.

### 3.2. Hydroxyl/rGO Combined with CSH Units in the Presence of Ca Ions and Hydroxyl Groups

#### 3.2.1. Hydroxyl/rGO with Silicate Hydrate Units in the Presence of Ca Ions

**Figure 4:**
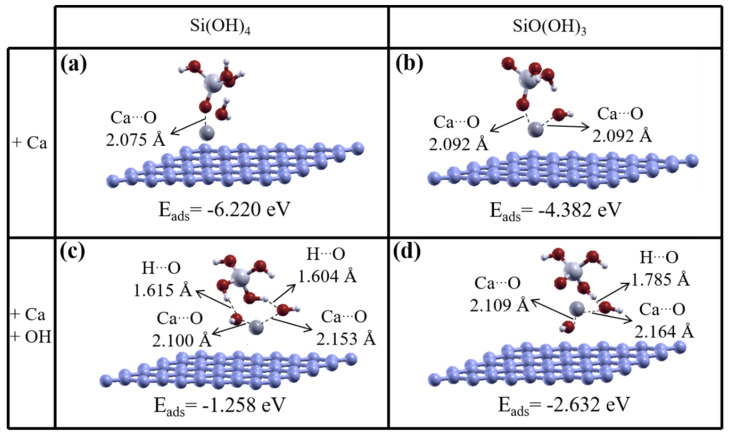
Optimized geometric structures including a silicate hydrate unit (Si(OH)_4_ or SiO(OH)_3_) and a Ca ion on a graphene plane, with the Ca ions initially close to the units on the hydroxyl/reduced graphene oxide sheet. The upper panels are for systems containing Ca ions; and the lower panels are for systems containing Ca ions and hydroxyl groups. The adsorption energies for SiO(OH)_3_ and Si(OH)_4_ are indicated beneath the respective configuration.

Comprehensive investigations of systems containing Si(OH)_4_ and hydroxyl/rGO sheets ending in SiO(OH)_3_ were performed. Previously studied structures, including Si(OH)_4_ and SiO(OH)_3_ units with a hydroxyl/rGO substrate, were used, but in the presence of Ca ions. Two different Si(OH)_4_ and hydroxyl/rGO configurations are used with a Ca ion at different positions, one with the Ca ion close to Si(OH)_4_ and the other with the Ca ion far from Si(OH)_4_, as the initial geometric structures. The structures were optimized, and the ground-state energy was found to be 0.495 eV lower for the configuration with a Ca ion close to Si(OH)_4_ (Figure 4a) than for the other initial configuration with a Ca ion far from Si(OH)_4_. In fact, for the same configuration without a Ca ion, charge transfer occurred from the graphene sheet to the 2p orbital of the dangling oxygen atom in SiO(OH)_3_. Therefore, in a system containing a Ca ion, the Ca ion interacts directly with SiO(OH)_3_. Some of the charge on the Ca ion is transferred to the 2p orbital of an oxygen atom in SiO(OH)_3_, to occupy the orbital fully, and the remaining charge is transferred to the graphene sheet. The structure of Si(OH)_4_ on a hydroxyl/rGO substrate was also optimized in the presence of a Ca ion, in a doubly positively charged system. The hydroxyl group dissociates from the hydroxyl/rGO substrate and a water molecule is formed, and the Ca ion also interacts with SiO(OH)_3_. The distance between the Ca ion and the oxygen atom in the ground-state structure is ~2.075 Å, as shown in Figure 4a, which was 0.1 Å more than the distance in the other configuration.

The results indicate that the adsorption energies of SiO(OH)_3_ in the four different configurations, with a water molecule on the graphene surface, vary between −1.43 and −1.69 eV; the latter is given as the ground state indicated in Figure 2b. These adsorption energies of the SiO(OH)_3_ model are slightly higher than the adsorption energies of −1.36 to −1.59 eV, when no water molecule is present on the graphene surface (Figure 3). The energies for the adsorption of SiO(OH)_3_ to the graphene surface are a few eV lower, at −6.22 eV to −4.38, in the presence of a water molecule or hydroxyl group, and a Ca ion (Figure 4a,b). In other words, the adsorption energy decreases when a Ca ion is added to the system.

When a Ca ion is far from SiO(OH)_3_ on the hydroxyl/rGO sheet in the initial simulation model, the hydroxyl group is found to not dissociate from the hydroxyl/rGO sheet. A lower energy, −1.93 eV, is found for the ground-state structure than in other configurations. However, the ground-state structure in the initial simulation model relates to the configuration with the Ca ion near SiO(OH)_3_. Optimization for the ground-state structure indicates that the hydroxyl group becomes dissociated from the hydroxyl/rGO, and the Ca ion is involved in bonding, so charge transfer from the Ca ion to two neighboring oxygen atoms occurs, to cause a nearly full occupation of the oxygen orbitals (Figure 4b).

#### 3.2.2. Hydroxyl/rGO with Silicate Hydrate Units, in the Presence of Ca Ions and Involving Hydroxyl Groups

We also studied the interactions between Si(OH)_4_ and SiO(OH)_3_, and the hydroxyl/rGO sheet, in the presence of a hydroxyl group and a Ca ion. The results indicate that the ground-state structure has the same configuration as the structure with a Ca ion close to the silicate monomer, which causes the hydroxyl group to dissociate from the hydroxyl/rGO sheet and the charge to be compensated by charge transfer from the Ca ion to the two neighboring oxygen atoms on two hydroxyl groups, as shown in Figure 4c,d. The ground-state structures of Si(OH)_4_ and SiO(OH)_3_ have energies that are almost 3 and 0.4 eV lower, respectively, than the next lowest energy configurations. In contrast, for the configuration with a Ca ion that is far from the silicate, the hydroxyl group is not dissociated from the sheet. The adsorption energy for SiO(OH)_3_ in the ground-state structure, in the presence of a hydroxyl group and a Ca ion on the graphene plane, is −2.63 eV, which indicates that stronger adsorption occurred than for the removal of only hydroxyl, as shown in Figure 4c. In fact, even when more hydroxyl groups are added to the system, the hydroxyl group becomes dissociated from the hydroxyl/rGO sheet, because the hydroxyl groups from Si(OH)_4_ remain strongly bonded to the Si atom.

### 3.3. Epoxide/rGO with CSH Units

**Figure 5:**
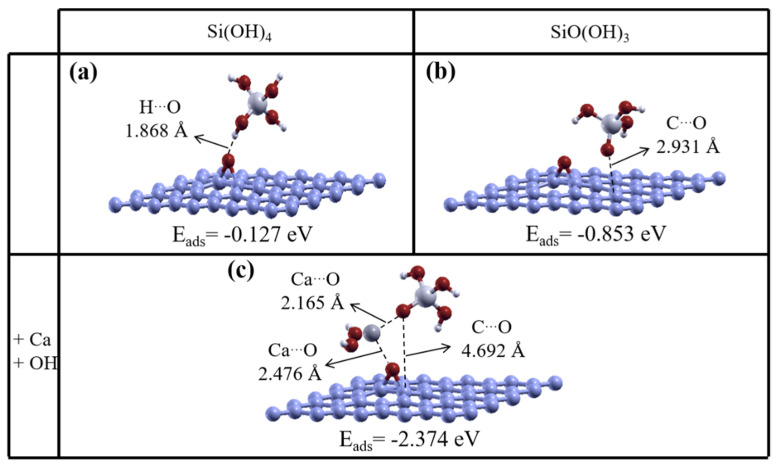
Optimized geometric structures of (calcium) silicate hydrate units on an epoxide/reduced graphene oxide (rGO) sheet. The adsorption energies are shown below each structure. Adsorption of a silicate unit to epoxide/rGO becomes more favorable in the presence of a Ca ion.

As mentioned above, the hydroxyl group in the ground-state structures of the CSH composites dissociates from the hydroxyl/rGO sheet, to produce pristine graphene (Figure 2b). According to previous works [70], the contribution of carbonyl/epoxy groups on nanocomposites is still important, and they are not fully hydrolyzed during the preparation of composites. However, as shown in Figure 5a, pristine graphene is not produced when Si(OH)_4_ is on the epoxide/rGO surface, and the ground-state structure remains similar to the initial structure, with E_ads_~0.127 eV (2.93 kcal/mol). The mechanism involved in the interaction between SiO(OH)_3_ and the surface of the epoxide/rGO sheet was investigated, and the results are shown in Figure 5b. We found an adsorption energy for SiO(OH)_3_ on the epoxide/rGO surface of −0.853 eV, which is lower than the adsorption energy for a neutral Si(OH)_4_ silicate hydrate moiety. The SiO(OH)_3_ silicate unit moves away from the epoxide groups, as shown by the physisorption model described above. The nearest distance between the dangling oxygen atom and the epoxide/rGO sheet is 2.931 Å. Even longer distances were found in several previous studies. For example, Gao et al. [71] calculated the adsorption energies for H_2_S and CH_4_ on intrinsic graphene, which were −0.038 and −0.022 eV, respectively, and distances of 3.813 and 3.865 Å, which are larger than the distances included in Figure 5. Then, the interaction between SiO(OH)_3_ and the epoxide/rGO sheet was considered to be neutral in the presence of a Ca ion and two hydroxyl groups, as shown in Figure 5c. As discussed above, the adsorption energy becomes more favorable when Ca ions are involved. The adsorption energy for SiO(OH)_3_, in the presence of a Ca ion and hydroxyl functional groups of hydroxyl, is therefore −1.52 eV lower (Figure 5c) than the adsorption energy for the same structure without the functional groups (Figure 5b).

## 4. Discussion

### 4.1. Electronic Properties of the Ground State with the Condensation Reaction

**Figure 6:**
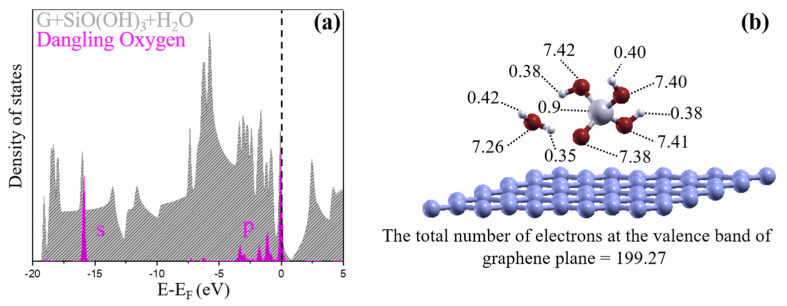
(**a**) Density of states and (**b**) charge distribution of the ground state for reduced graphene oxide with hydroxyl groups in a water molecule and a SiO(OH)_3_ moiety on the graphene plane. The Fermi level is shown as a dashed line at the value of zero. The partial density of states on the unprotonated oxygen is shown in pink. The charges were associated with the atoms by Bader charge analysis.

The electronic properties of the ground-state structures described above are now analyzed in more detail. The total DOS of the ground-state structure consisting of a graphene layer, a water molecule, and a SiO(OH)_3_ unit is shown in Figure 6a. The Fermi level is indicated by a vertical dashed line at the value of zero. The charge neutrality point for the graphene layer was higher than the Fermi level, because the graphene layer was positively charged. The DOS that was projected on the non-protonated oxygen atom belonging to SiO(OH)_3_ is plotted in pink. Below the Fermi level, at the valence band energy of −0.073 eV, there is a large DOS peak from the oxygen atom with nearly one extra electron. Bader charge analysis shows the charge density distribution assigned to atoms, as shown in Figure 6b. Thus, our results indicate that there is charge transfer from graphene to the dangling oxygen atom, in order to have the amount of electrons at the valence fully occupied for the SiO(OH)_3_ moiety.

### 4.2. Electronic Properties of the Ground State SiO(OH)_3_ with the Graphene Sheet

**Figure 7:**
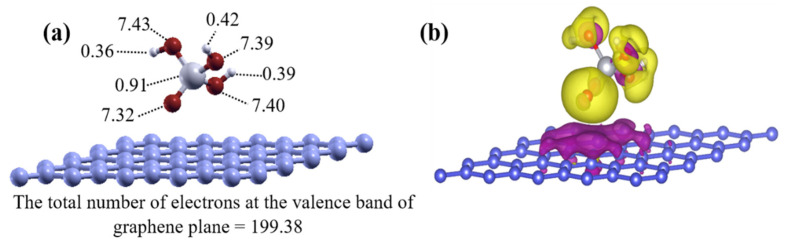
(**a**,**b**) Charge density distributions for the neutral model with the SiO(OH)_3_ unit physisorbed to graphene determined by Bader charge analysis and the spatial distribution of the three-dimensional charge density differences. The yellow and purple isosurfaces indicate charge gains and losses, respectively. Almost one electron is transferred to SiO(OH)_3_ from the graphene layer to establish the strong bond, which is stabilized further by a water molecule, as shown by the adsorption energies (see discussion in the text).

The electronic properties of the ground-state structures given by the physisorption model of SiO(OH)_3_ on the graphene plane, for the neutral and singly negatively charged systems, were assessed. The Bader charge analysis method indicates that the charge transfers between the graphene and SiO(OH)_3_ were similar to the charge transfers in the ground state on the rGO, as shown in Figure 7a. For the neutral system, the graphene sheet lost 0.62 electrons and the charge density of the dangling oxygen became 7.32 electrons (Figure 7a).

We constructed the 3D charge density difference plots to determine the spatial distribution of the charge. The charge density differences for the ground-state structure of SiO(OH)_3_ on the graphene plane in the neutral system, with respect to the graphene sheet (200 electrons) and the SiO(OH)_3_ unit (31 electrons), are shown in Figure 7b. The distribution of electrons between the substrate and absorbent matched the results of the Bader charge analysis, because an electron was transferred to the SiO(OH)_3_ unit. The adsorption energy for SiO(OH)_3_ on graphene was calculated with respect to the negative unit, and was in the range of a few electron volts. This is typical for Coulomb interactions over a few angstroms distance, caused by charge transfer. Water molecules near the graphene increase the adsorption energy because they form hydrogen bonds with SiO(OH)_3_, and because the oxygen atom in a water molecule becomes more stable by interacting with the depleted positive charge in the graphene layer.

### 4.3. Electronic Properties of the Ground State with Condensation Reaction after Addition of a Ca Ion

**Figure 8:**
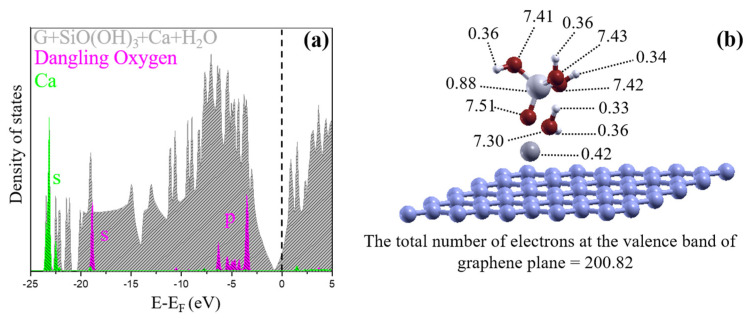
(**a**) Density of states and (**b**) charge distribution of the ground state found for rGO with hydroxyl groups, which is compound of a water molecule, a SiO(OH)_3_ unit and a Ca ion on the graphene plane. The Fermi level is marked by dashed line and assigned to zero. The partial density of states on the dangling oxygen and calcium are also included using magenta and green colors, respectively. The charges are associated to atoms using Bader charge analysis. Note that because of the Ca ion, the graphene layer is doped negatively. The unprotonated oxygen receives more charge and the silicate hydrate unit is deposited above the Ca ion on the graphene.

We assessed the changes in the hydroxyl/rGO electronic properties, caused by adding CSH. The structures with the most favorable adsorption energies were analyzed. The DOS and Bader charge distributions are shown in Figure 8. The DOS indicated that the graphene Fermi level moves above the neutrality point, i.e., into the linear part above the density of states of zero. The oxygen and calcium states are not near the Fermi level, and are more than 1 eV from the graphene neutrality point, meaning that these states are almost fully occupied and empty, respectively. Although the neutrality point can be recovered by doping, occupation of the CSH counterpart remains similar, even for the cases involving hydroxyl groups. The unprotonated oxygen atom has more charge than the neutral cases discussed above, and it becomes almost equally charged as the case for the negatively charged systems. The Ca ion on graphene is almost unoccupied, but has a charge of 0.42 electrons because it is close to graphene. This value is almost the same independently as for the Ca configuration, as long as the Ca ion remains close to the graphene. It seems that the Ca ion helps to form a sandwich structure of charges between the graphene layer and silicate hydrate unit, giving very favorable adsorption energies.

### 4.4. Electronic Properties of the Ground-State Epoxide/rGO with the Silicate Hydrate Unit

**Figure 9:**
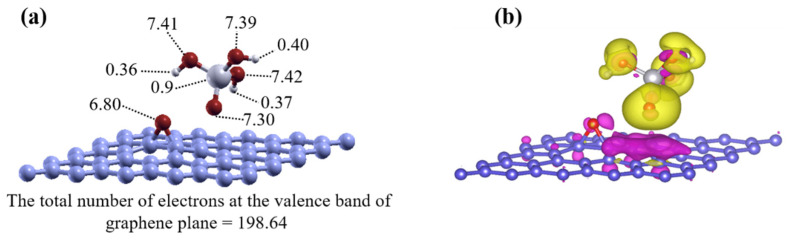
(**a**) Charge density distribution for SiO(OH)_3_ on epoxide/reduced graphene oxide determined by Bader charge analysis. (**b**) Three-dimensional charge density differences for the neutral system. Yellow and purple isosurfaces indicate charge gains and losses, respectively.

The charge density distribution of the neutral systems, determined by Bader charge analysis, and the charge density differences for the molecular orbital isosurfaces, are shown in Figure 9. The Bader charge analysis, the results of which are shown in Figure 9a, indicated that there are 7.30 electrons on the unprotonated oxygen atom. The addition of an electron causes the electron density of the dangling oxygen atom to increase slightly, from 7.30 to 7.46, 0.73 electrons to be transferred to the graphene plane, and 0.11 electrons to be shared between the other atoms. Three-dimensional charge density difference plots were produced, giving more details of the distributions of electrons between the substrate and absorbents than were given by the Bader charge analysis, in order to investigate the electron distributions further. The charge density differences for the ground-state structure of SiO(OH)_3_ on the graphene plane in the neutral system are shown in Figure 9b. Graphene lost charge to the silicate hydrate unit because it was far from the epoxide group. In fact, the bond between graphene and the SiO(OH)_3_ unit resembles the bond in the basic bonding model described in Figure 3 and Figure 6. In other words, the adsorption energies indicated that the charge transfer from the graphene plane to SiO(OH)_3_ occurs in the neutral system, to give an almost fully occupied oxygen 2p orbital.

## 5. Conclusions

A DFT method was used to study the mechanism involved in the interactions between hydroxyl or epoxide rGO and the CSH moieties, such as CSH gel in cement. The DFT calculations for silicate tetrahedra, Ca ions, and hydroxyl groups improve our understanding of the bonds between rGO and primary CSH moieties. The results led to the following conclusions. The interactions between hydroxyl/rGO and silicate tetrahedra can repair hydroxyl defects selectively in the rGO lattice, and cause graphene to re-form. The dissociation of defects in the graphene plane, and the formation of water, even occurs in the presence of Ca ions and hydroxyl groups. In fact, the main interactions between the graphene plane and CSH gel are Coulomb interactions, caused by charge transfer. In contrast, the ground-state structure remains similar to the initial structure model for interactions between epoxide/rGO and CSH gel. Consideration of the strong interactions in this way could allow improvements to be made in the design of composite materials.

## Data Availability

Data are contained within the article.
